# Trends in heterosexual inexperience among young adults in Japan: analysis of national surveys, 1987–2015

**DOI:** 10.1186/s12889-019-6677-5

**Published:** 2019-04-08

**Authors:** Cyrus Ghaznavi, Haruka Sakamoto, Daisuke Yoneoka, Shuhei Nomura, Kenji Shibuya, Peter Ueda

**Affiliations:** 1 Department of Global Health Policy, Graduate School of Medicine, The University of Tokyo, Tokyo, 113–0033 Japan; 2 Clinical Epidemiology Division, Department of Medicine, Solna, Karolinska Institutet, 17071 Stockholm, Sweden

**Keywords:** Japan, Sexual experience, Sexual inexperience, Sexual inactivity, Virginity

## Abstract

**Background:**

It has been suggested that an increasing number of Japanese adults remain sexually inexperienced; however, no study has assessed this issue using nationally representative data.

**Methods:**

We used data from seven rounds of the National Fertility Survey of Japan, 1987–2015, and included adults aged 18–39 years (18–34 years in the 1987 survey) in the analyses (sample size 11,553–17,850 [1987–2010]; response rate 70.0–92.5%). For each survey year, sex and age group, we estimated the age-adjusted prevalence of heterosexual inexperience, defined as reporting no experience of sexual intercourse with someone of the opposite sex. We used logistic regression, adjusted for age, to identify factors associated with heterosexual inexperience in the 2010 survey. Information about same-sex sexual experience was not available.

**Results:**

Between 1992 and 2015, the age-standardized prevalence of heterosexual inexperience in adults aged 18–39 years increased from 21.7 to 24.6% for women (*p*-values for linear and quadratic trend < 0.05) and from 20.0 to 25.8% for men (*p*-values for trend < 0.05). Among those aged 30–34 years, the prevalence was 6.2% in 1987 and 11.9% in 2015 for women (*p*-values for trend ≥0.05) and 8.8% (1987) and 12.7% (2015) for men (p-values for trend ≥0.05). Among those aged 35–39 years, prevalence increased from 4.0% in 1992 to 8.9% in 2015 among women (p-values for trend < 0.05). The corresponding numbers for men in the same age group were 5.5 and 9.5%, respectively (p-values for trend ≥0.05). Among men aged 25–39 years, unemployment, temporary/part-time employment and lower income were associated with heterosexual inexperience.

**Conclusions:**

The proportion of young Japanese adults with no experience of heterosexual intercourse had increased in the past two decades. Among adults in their thirties, around one in ten had no heterosexual experience. Unemployment, temporary/part-time employment and low income were associated with heterosexual inexperience among men. Further research is needed on the factors contributing to and the potential public health and demographic implications of the high proportion of the Japanese population that remains sexually inexperienced well into adult age.

**Electronic supplementary material:**

The online version of this article (10.1186/s12889-019-6677-5) contains supplementary material, which is available to authorized users.

## Background

Japan’s total fertility rate is among the lowest in the world, and the Japanese population is predicted to fall by a third by 2060 [1]. While declines in stable employment opportunities and difficulties combining full-time work with child-rearing are commonly mentioned as explanations for the low birth rates, [2] it has also been suggested that a decline in sexual activity among the country’s young adults has contributed to the demographic decline [3–7].

In the 2015 National Fertility Survey, more than 40% of never-married 18–34-year-old women and men responded that they have never had sexual intercourse with someone of the opposite sex [8]. Comparisons with previous surveys indicated that this proportion had increased in recent years [8]. Although many sexually active individuals do not have children and some women may conceive without a male sexual partner, heterosexual intercourse is the main method of human reproduction; consequently, sexual inexperience in a large part of the population may affect fertility rates. Moreover, sexual health and satisfaction are key components of human health, well-being and life satisfaction [9–12]. The purported increase in individuals who remain sexually inexperienced well into adulthood has become a national concern [3–7] and may have important demographic and public health implications.

Important knowledge gaps regarding sexual inexperience among young Japanese adults remain. Previous analyses have been limited to never-married individuals; thus, the prevalence of heterosexual inexperience in the whole population, also including those who are married, is unknown. In addition, prevalence of sexual inexperience among never-married individuals is not comparable across years as the average age of marriage and marriage rates have changed over the past decades [13]. Accordingly, the size and potential changes over time of the proportion of the population that is heterosexually inexperienced have not been investigated.

In this study, we used nationally representative data from seven rounds of the National Fertility Survey, 1987 to 2015, to estimate age-standardized prevalence of heterosexual inexperience in the general population, investigate the number and characteristics of inexperienced individuals, and assess socioeconomic and regional factors associated with heterosexual inexperience.

## Methods

### Data sources

We used data from rounds 9 to 15 of the National Fertility Survey, conducted in 1987, 1992, 1997, 2002, 2005, 2010, and 2015. The survey is carried out by the National Institute of Population and Social Security Research (IPSS), under the Japanese Ministry of Health, Labour, and Welfare, to collect nationally representative data on topics related to marriage and child birth in Japan, [8, 14] as described in Additional file 1. In brief, each survey used stratified cluster sampling with districts in the Population Census of Japan as primary sampling units and comprised two national sub-surveys: one for married couples in which the wife was under 50 years of age, and one for unmarried women and men between 18 and 49 years of age (except the 1987 survey of unmarried adults which only included those aged 18–34 years) [8]. Through home visits, participants were provided with a self-administered questionnaire which was returned upon completion in a sealed envelope at a follow-up visit. The response rate across survey years ranged between 70.0 and 83.8% among unmarried individuals and between 85.7 and 92.5% among the married couples [8].

Use of data on stratification and primary sampling units was not approved by IPSS due to constraints on secondary data usage. As these variables are needed to calculate confidence intervals that account for the stratified cluster sampling, confidence intervals in our analyses should be interpreted with caution. (Additional file 1) We obtained information about the number of individuals in the Japanese population by age, sex and marital status from the Population Census of Japan (1985–2015) [13]; these data were used to weight the sample to ensure that it was broadly representative of the Japanese population with respect to sex, age and marital status, as described below.

### Study population

We used individual-level data from the surveys conducted between 1987 and 2010 and summary data from the 2015 survey report published by IPSS [8] as use of individual-level data in the 2015 survey was not approved by IPSS. We included married and unmarried women and men, aged between 18 and 39 years. We excluded unmarried participants with unknown status of heterosexual experience as defined below. Across the surveys from 1987 to 2010, the proportion of those excluded ranged from 2 to 7% (5 to 12% of the unmarried participants) among women and from 2 to 7% (4 to 11% of the unmarried participants) among men. The characteristics of excluded participants were largely similar to the unmarried participants with known heterosexual experience status who were included in the analyses (Additional file 1: Tables S1 and S2). The final sample size of the surveys from 1987 to 2010 ranged between 11,553 (5787 unmarried) and 17,850 (8243 unmarried). (Additional file 1: Table S3).

We used sample weights to adjust for differential probabilities of non-response and unknown status of heterosexual experience by age, sex and marital status. Sample weights were calculated as the inverse of the probability of being sampled according to age, sex and marital status based on data from the Population Census of Japan [13] as described in the Additional file 1.

### Definition of sexual inexperience

The main outcome was heterosexual inexperience (yes/no). We assumed that all married participants and unmarried participants who had been previously married had heterosexual experience [15]. The sub-survey of unmarried participants included the question, “Have you ever had sexual intercourse with someone of the opposite sex?” Never-married participants who answered “no” to this question were categorized as sexually inexperienced and those who answered “yes” were categorized as having heterosexual experience. The wording of the question was consistent in all surveys; in the 2005 and 2015 surveys, the question was asked together with questions about contraception and in the 2005 survey, participants could specify whether they had experience from intercourse within the past year. (Additional file 1: Table S4) The Japanese term for sexual intercourse (*seikosho*) that was included in the survey is commonly used to refer to vaginal intercourse. The survey did not contain questions about same-sex sexual experience, and same-sex marriage is not established in Japan; our analyses were therefore limited to heterosexual intercourse.

### Statistical methods

All analyses were stratified by sex and performed in Stata version 14.0 (StataCorp LP, College Town, TX) and R version 3.3.2. For each survey from 1987 to 2010, we used sample weights that were standardized to the age distribution of 2015, as described in Additional file 1, to estimate age-standardized prevalence of heterosexual inexperience for the full age range (18–39 years) and by age group (18–19; 20–24; 25–29; 30–34; and 35–39 years) in the total and never-married populations. Prevalence for ages 18–39 years and 35–39 years were not estimated for 1987 as this survey did not include unmarried participants older than 34 years. Because individual level data were not available for the 2015 survey, we used summary data provided in the survey report [8] and marital status data taken from the 2015 Population Census of Japan to estimate the prevalence, as described in Additional file 1. These estimates were directly comparable to those of the years 1987–2010 as they were based on the same survey question and census data (as shown in Additional file 1); however, confidence intervals could not be calculated. Linear and quadratic trends in prevalence across the survey years were assessed using linear regression, for which *p*-values of less than 0.05 were regarded as statistically significant.

Next, we determined the proportion of heterosexually inexperienced women and men using the 2010 survey (the most recent survey for which individual-level data were available). We applied sample weights (without age-standardization, as described in Additional file 1) to extrapolate population characteristics, by heterosexual experience status, to Japan’s 16.33 million women and 16.86 million men, aged 18–39 years in 2010 [13]. We selected, a priori, variables that we hypothesized could be associated with sexual inexperience: education, occupational status, annual income, region of residence and population size/density of residence [16–18]; the definitions and categorizations of these variables are shown in Additional file 1: Table S8. Among those who were sexually inexperienced, we calculated the proportion who responded that they desire marriage in their lifetime.

Finally, we analysed the association between the selected variables and heterosexual inexperience using 2010 survey data. We employed logistic regression, accounting for sample weights, to calculate age-adjusted odds ratios (aORs), with heterosexual inexperience as the binary dependent variable (1 = heterosexually inexperienced; 0 = heterosexually experienced). We adjusted for age as a continuous variable. We conducted the analyses by age group (18–24 years and 25–39 years) because associations between the assessed variables and sexual inexperience are likely to differ by age. Participants with missing data on the investigated variables were few and excluded from the analyses by variable: education (weighted proportion 1.1%), occupational status (4.2%), annual income (3.2%) and desire for marriage during the lifetime (4.6% among those with no heterosexual experience). The study was approved by the Regional Ethics Committee at The University of Tokyo, Japan. Informed consent was not required for secondary use of data from the National Fertility Survey.

## Results

### Age-standardized prevalence of heterosexual inexperience, 1987–2015

**Fig. 1:**
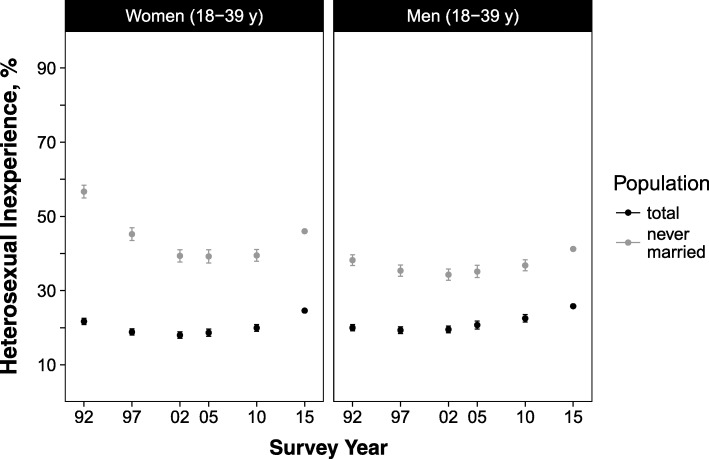
Age-standardized prevalence of heterosexual inexperience in Japanese women and men, aged between 18 and 39 years, 1992–2015. Footnote: Prevalence in 1992–2010 was standardized to the age distribution of 2015. Error bars represent 95% confidence intervals. Data are shown in Additional file 1: Tables S9 and S10

**Fig. 2:**
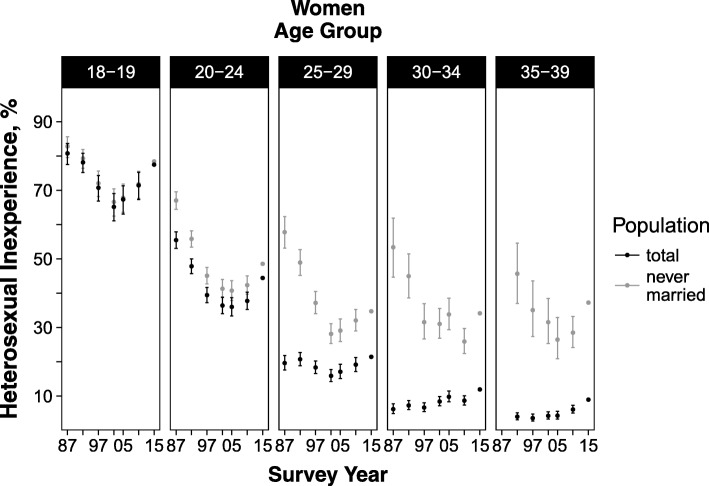
Age-standardized prevalence of heterosexual inexperience in Japanese women by age group, 1987–2015. Footnote: Prevalence in 1987–2010 was standardized to the age distribution of 2015. Prevalence for those aged 35–39 years was not presented for 1987 as this survey did not include unmarried participants older than 34 years. Error bars represent 95% confidence intervals. Data are shown in Additional file 1: Table S9

The overall age-standardized prevalence of sexual inexperience among women aged 18–39 years had increased from 21.7% in 1992 to 24.6% in 2015 (*p*-values for linear and quadratic trend < 0.05). (Fig. 1 and Additional file 1: Table S9) When analysed by age group, the highest prevalence was observed in 1987 among those aged 18–19 years (80.7%) and 20–24 years (55.5%); the prevalence then decreased, reaching its lowest levels in 2002 (18–19 years, 65.1%) and 2005 (20–24 years, 36.0%), at which point it increased again (prevalence in 2015: 18–19 years, 77.5%; 20–24 years, 44.4%; *p*-values for trend < 0.05). In those aged 30–34 years, prevalence almost doubled during the study period, from 6.2% in 1987 to 11.9% in 2015, although this increase was not statistically significant (*p*-values for trend ≥0.05). Similarly, in those aged 35–39 years, prevalence increased from 4.0% in 1992 to 8.9% in 2015 (p-values for trend < 0.05). (Fig. 2 and Additional file 1: Table S9).

**Fig. 3:**
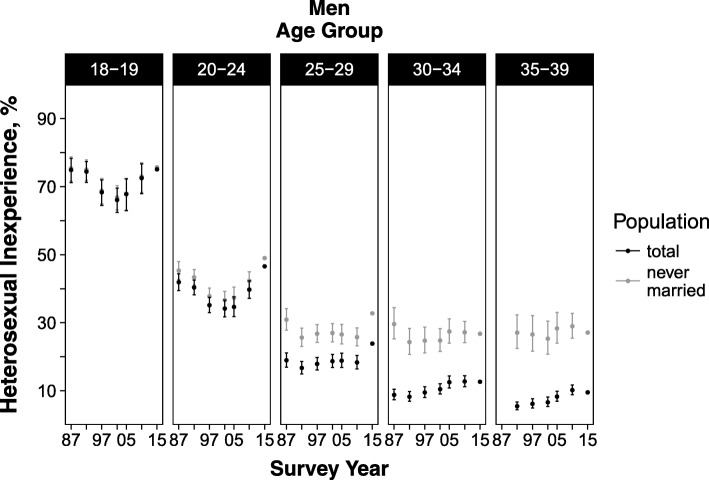
Age-standardized prevalence of heterosexual inexperience in Japanese men by age group, 1987–2015. Footnote: Prevalence in 1987–2010 was standardized to the age distribution of 2015. Prevalence for those aged 35–39 years was not presented for 1987 as this survey did not include unmarried participants older than 34 years. Error bars represent 95% confidence intervals. Data are shown in Additional file 1: Table S10

Among men, the age-standardized prevalence in those aged 18–39 years had increased from 20.0% in 1992 to 25.8% in 2015 (*p*-values for linear and quadratic trend < 0.05). (Fig. 1 and Additional file 1: Table S10) In the younger age groups, prevalence decreased between 1987 (18–19 years, 74.9%; 20–24 years, 41.9%) and 2002 (18–19 years, 66.1%; 20–24 years, 34.2%), after which it increased (prevalence in 2015: 18–19 years, 75.1%; 20–24 years, 46.6%; *p*-values for trend < 0.05). (Fig. 3 and Additional file 1: Table S10) In the oldest age groups, prevalence rose nominally during the study period: from 8.8% in 1987 to 12.7% in 2015 among those aged 30–34 years (p-values for trend ≥0.05), and from 5.5% in 1992 to 9.5% in 2015 among those aged 35–39 years (p-values for trend ≥0.05). (Fig. 3 and Additional file 1: Table S10).

### Population characteristics and age-adjusted odds ratios in 2010

The characteristics of the participants in the 2010 National Fertility Survey are shown in Additional file 1: Table S11, and the sample-weighted extrapolation of the results to the Japanese population are shown in Table 1 (18–39 years), Additional file 1: Table S12 (18–24 years) and Additional file 1: Table S13 (25–39 years). Mean (SD) age was 30.0 (6.3) years for women and 30.0 (6.2) years for men, and 44.4% of women and 36.6% of men were married. A larger proportion of women (25.3%) than men (6.6%) were unemployed while more men (61.7%) than women (32.3%) were regular employees. In total, 3.26 million women and 3.80 million men, aged 18–39 years, were estimated in 2010 to have no heterosexual experience. (Table 1) Of those without heterosexual experience, 85.8% of the women and 82.8% of the men responded that they desire marriage during their lifetimes. (Table 1) Among those without heterosexual experience aged 25–39, this proportion was 81.0% for women and 78.2% for men.

**Table 1:** Extrapolation of the characteristics of adults, aged 18 to 39 years, from the 2010 National Fertility Survey to all Japanese adults aged 18 to 39 years, by sex and heterosexual experience status. Numbers are shown in 1000s

	Women (*N* = 16,330)			Men (N = 16,860)		
	All	Heterosexual experience		All	Heterosexual experience	
		No (*n* = 3256 [19.9%])	Yes (*n* = 13,074 [80.1%])		No (*n* = 3795 [22.5%])	Yes (n = 13,065 [77.5%])
	n (%)	n (%)	n (%)	n (%)	n (%)	n (%)
Age						
18–19	1145 (7.0)	817 (25.1)	327 (2.5)	1210 (7.2)	877 (23.1)	327 (2.5)
20–24	3034 (18.6)	1146 (35.2)	1883 (14.4)	3143 (18.6)	1249 (32.9)	1894 (14.5)
25–29	3457 (21.2)	661 (20.3)	2798 (21.4)	3555 (21.1)	653 (17.2)	2900 (22.2)
30–34	3985 (24.4)	345 (10.6)	3635 (27.8)	4105 (24.3)	524 (13.8)	3580 (27.4)
35–39	4710 (28.8)	287 (8.8)	4419 (33.8)	4846 (28.7)	493 (13.0)	4351 (33.3)
Marital Status						
Unmarried	9071 (55.6)	3256 (100)	5818 (44.5)	10,695 (63.4)	3795 (100)	6898 (52.8)
Married	7259 (44.4)	0 (0)	7256 (55.5)	6165 (36.6)	0 (0)	6167 (47.2)
Education^a^						
High school or less	6126 (37.5)	1031 (31.7)	5087 (38.9)	7471 (44.3)	1645 (43.3)	5827 (44.6)
Vocational school or short college	5930 (36.3)	978 (30.0)	4955 (37.9)	2880 (17.1)	614 (16.2)	2268 (17.4)
Undergraduate studies	4020 (24.6)	1194 (36.7)	2834 (21.7)	5672 (33.6)	1386 (36.5)	4285 (32.8)
Graduate studies	254 (1.6)	53 (1.6)	198 (1.5)	837 (5.0)	151 (4.0)	686 (5.2)
Occupational Status						
Regular employee	5280 (32.3)	1065 (32.7)	4213 (32.2)	10,401 (61.7)	1256 (33.1)	9129 (69.9)
Part-time or temporary worker	4625 (28.3)	778 (23.9)	3848 (29.4)	1981 (11.7)	612 (16.1)	1367 (10.5)
Business owner or family business	559 (3.4)	51 (1.6)	501 (3.8)	1244 (7.4)	121 (3.2)	1121 (8.6)
Unemployed	4138 (25.3)	304 (9.3)	3834 (29.3)	1119 (6.6)	535 (14.1)	588 (4.5)
Student	1728 (10.6)	1058 (32.5)	677 (5.2)	2115 (12.5)	1272 (33.5)	861 (6.6)
Annual Income (in JPY 10,000s)^b^						
0–99	9057 (55.5)	1920 (59.0)	7144 (54.6)	5005 (29.7)	2333 (61.5)	2675 (20.5)
100–299	4821 (29.5)	981 (30.1)	3841 (29.4)	4092 (24.3)	906 (23.9)	3189 (24.4)
300–499	2101 (12.9)	321 (9.9)	1779 (13.6)	5468 (32.4)	485 (12.8)	4986 (38.2)
500–799^c^	351 (2.1)	34 (1.0)	310 (2.4)	1992 (11.8)	67 (1.8)	1919 (14.7)
≥ 800	–	–	–	303 (1.8)	4 (0.1)	297 (2.3)
Region of Residence^d^						
Kanto	5455 (33.4)	1123 (34.5)	4327 (33.1)	5617 (33.3)	1355 (35.7)	4259 (32.6)
Chubu	3277 (20.1)	632 (19.4)	2641 (20.2)	3379 (20.0)	778 (20.5)	2600 (19.9)
Kinki	2531 (15.5)	596 (18.3)	1935 (14.8)	2486 (14.7)	607 (16.0)	1881 (14.4)
Kyushu/Okinawa	1891 (11.6)	316 (9.7)	1582 (12.1)	2016 (12.0)	376 (9.9)	1646 (12.6)
Chugoku/Shikoku	1377 (8.4)	274 (8.4)	1098 (8.4)	1496 (8.9)	304 (8.0)	1189 (9.1)
Tohoku	1247 (7.6)	218 (6.7)	1033 (7.9)	1328 (7.9)	300 (7.9)	1032 (7.9)
Hokkaido	553 (3.4)	101 (3.1)	458 (3.5)	539 (3.2)	80 (2.1)	457 (3.5)
Area of residence: population size and density						
Non-densely inhabited area	5498 (33.7)	1146 (35.2)	4354 (33.3)	5732 (34.0)	1309 (34.5)	4429 (33.9)
< 200,000	3492 (21.4)	700 (21.5)	2798 (21.4)	3725 (22.1)	827 (21.8)	2900 (22.2)
200,000 to < 1,000,000	3996 (24.5)	729 (22.4)	3269 (25.0)	4096 (24.3)	941 (24.8)	3162 (24.2)
≥ 1,000,000	3344 (20.5)	681 (20.9)	2667 (20.4)	3307 (19.6)	721 (19.0)	2587 (19.8)
Wish to get married in lifetime						
No	–	461 (14.2)	–	–	653 (17.2)	–
Yes	–	2795 (85.8)	–	–	3142 (82.8)	–

^a^Currently enrolled students were categorized according to their ongoing education

^b^Income was categorized according to the individual’s revenues. 10,000 JPY was approximately 78 Euro as of June 2018

^c^≥ JPY 500 for women

^d^The seven regions constitute geographically clustered prefectures (the highest administrative divisions of Japan) and are often used in discussion of regional economic and policy issues in the country. The regions are shown by population size

JPY, Japanese Yen

In the logistic regression analyses, several factors associated with heterosexual inexperience were identified. (Table 2) For example, among men aged 25–39 years, part-time or temporary employment (aOR vs. regular employee, 3.82 [95% CI, 3.04 to 4.80]) and unemployment (aOR, 7.87 [95% CI, 6.06 to 10.23]) were associated with a higher likelihood of being heterosexually inexperienced. Conversely, among women, unemployment was associated with a lower likelihood of having no heterosexual experience. While the aOR for heterosexual inexperience decreased with each increase in annual income category among men, the aOR was lowest in women with the least income. In both sexes, the highest aORs for heterosexual inexperience were observed for those residing in the Kanto, Kinki and Chubu regions, while the lowest aORs were in Kyushu/Okinawa and Hokkaido. Compared to non-densely inhabited areas, residence in a densely inhabited area with over 1 million inhabitants was associated with a lower likelihood of heterosexual inexperience for men. The directions of the associations were largely similar among 18–24-year-olds, although there was no trend of a higher likelihood of heterosexual inexperience among women with higher income, and the Kanto (men and women), Hokkaido (women) and Chugoku/Shikoku (women) regions had the highest aORs.

**Table 2:** Age-adjusted odds ratios (aORs) for heterosexual inexperience and socioeconomic and regional factors among Japanese women and men aged 18 to 24 years and 25 to 39 years in 2010

	18–24 years		25–39 years	
	Women	Men	Women	Men
	aOR (95% CI)^a^	aOR (95% CI)^a^	aOR (95% CI)^a^	aOR (95% CI)^a^
Education^b c^				
High school or less	–	–	Ref	Ref
Vocational school or short college	–	–	1.10 (0.88 to 1.38)	0.86 (0.68 to 1.08)
Undergraduate studies	–	–	1.60 (1.26 to 2.02)	0.69 (0.57 to 0.84)
Graduate studies	–	–	2.40 (1.39 to 4.14)	0.77 (0.53 to 1.11)
p			< 0.001	0.001
Occupational Status				
Regular employee	Ref	Ref	Ref	Ref
Part-time or temporary worker	1.19 (0.90 to 1.57)	1.55 (1.09 to 2.20)	0.86 (0.69 to 1.07)	3.82 (3.04 to 4.80)
Business owner or family business	0.85 (0.30 to 2.41)	1.17 (0.52 to 2.63)	0.61 (0.36 to 1.05)	0.94 (0.64 to 1.36)
Unemployed	0.68 (0.47 to 0.99)	3.21 (2.07 to 4.99)	0.36 (0.27 to 0.47)	7.87 (6.06 to 10.23)
Student	1.58 (1.22 to 2.05)	1.90 (1.49 to 2.42)	1.19 (0.43 to 3.29)	4.76 (2.51 to 9.03)
p	0.014	< 0.001	< 0.001	< 0.001
Annual Income (in JPY 10,000s)				
0–99	Ref	Ref	Ref	Ref
100–299	0.78 (0.62 to 0.98)	0.56 (0.44 to 0.72)	2.19 (1.76 to 2.72)	0.49 (0.39 to 0.60)
300–499^d^	0.92 (0.57 to 1.50)	0.35 (0.23 to 0.51)	1.86 (1.42 to 2.43)	0.20 (0.16 to 0.25)
500–799^e^	–	–	1.95 (1.05 to 3.59)	0.09 (0.06 to 0.13)
≥ 800	–	–	–	0.05 (0.01 to 0.19)
	0.114	< 0.001	< 0.001	< 0.001
Region of Residence				
Kanto	Ref	Ref	Ref	Ref
Chubu	0.70 (0.54 to 0.91)	0.91 (0.69 to 1.20)	1.00 (0.77 to 1.29)	0.84 (0.67 to 1.07)
Kinki	0.90 (0.67 to 1.21)	0.63 (0.47 to 0.87)	1.41 (1.08 to 1.83)	1.15 (0.90 to 1.47)
Kyushu/Okinawa	0.68 (0.50 to 0.94)	0.60 (0.43 to 0.82)	0.69 (0.49 to 0.98)	0.61 (0.45 to 0.83)
Chugoku/Shikoku	0.99 (0.68 to 1.43)	0.71 (0.49 to 1.05)	0.91 (0.64 to 1.29)	0.75 (0.55 to 1.04)
Tohoku	0.68 (0.46 to 0.99)	0.69 (0.46 to 1.05)	0.86 (0.58 to 1.27)	1.00 (0.73 to 1.38)
Hokkaido	0.93 (0.56 to 1.55)	0.63 (0.36 to 1.11)	0.67 (0.37 to 1.21)	0.37 (0.19 to 0.71)
p	0.209	0.001	0.091	0.011
Population Density and Size of Residence				
Non-densely inhabited area	Ref	Ref	Ref	Ref
< 200,000	1.05 (0.81 to 1.35)	1.04 (0.79 to 1.37)	0.97 (0.75 to 1.24)	0.89 (0.71 to 1.11)
200,000 to < 1,000,000	0.77 (0.60 to 1.00)	0.97 (0.74 to 1.25)	0.95 (0.75 to 1.20)	1.03 (0.83 to 1.28)
≥ 1,000,000	0.94 (0.73 to 1.22)	1.01 (0.77 to 1.33)	0.81 (0.62 to 1.06)	0.74 (0.58 to 0.95)
p	0.257	0.928	0.144	0.093

^a^Adjusted for age as a continuous variable. Heterosexual inexperience was coded as 0 = has experience; 1 = has no experience

^b^Currently enrolled students were categorized according to their ongoing education

^c^Education was not analyzed in 18–24 year-old women and men because most adults in younger age groups may not have concluded their studies

^d^≥ JPY 300 for 18–24-year-olds

^e^≥ JPY 500 for 25–39-year old women

*aOR* age-adjusted odds ratio, *JPY* Japanese Yen

## Discussion

Sexual activity is a key component of human fertility and well-being [9–12]. In the face of low fertility rates and a shrinking population, sexual inexperience among young adults in Japan has been subject to concern and speculation [3–6]. Our study expands on available data on heterosexual inexperience in Japan by providing national estimates of prevalence by age group and by assessing socioeconomic and regional factors associated with having no heterosexual experience.

Between 1992 and 2015, age-standardized prevalence of heterosexual inexperience had increased among Japanese adults aged 18–39 years. In 2015, prevalence was 11.9% (women) and 12.7% (men) in those aged 30–34 years, and 8.9% (women) and 9.5% (men) in those aged 35–39 years; these numbers translate to 1.56 million adults in their thirties who had never engaged in heterosexual intercourse.

While national surveys on sexual behaviour in other high-income countries in Asia are scarce, data on sexual inexperience are available for some non-Asian high-income countries, e.g., the UK, [19] the US, [20] Australia, [18] Denmark [21] and New Zealand [22]. Compared to adults in these countries, [18–22] our results indicate that Japanese adults tend to become sexually active later in life and that a substantially larger proportion remain heterosexually inexperienced into their thirties. For example, in the nationally representative Natsal-3 study in the UK, (2011, 19] the proportion of women who reported no sexual partners of the opposite sex (defined as someone with whom the survey participant had engaged in vaginal, oral or anal intercourse) over the lifetime was 19.8% (16–24 years), 2.6% (25–34 years) and 0.5% (35–44 years); the corresponding numbers for men were 19.8, 5.2 and 1.5% [19]. In the 2006–2008 National Survey of Family Growth in the US, [20] the proportion of women reporting no sexual partners of the opposite sex after the age of 18 years were 12.6% (20–24 years), 3.4% (25–29 years), 1.9% (30–34 years) and 0.9% (35–39 years); the corresponding numbers for men were 14.4, 3.8, 3.1 and 1.4% [20]. In a nationally representative survey in Australia (2012–2013), the proportion of the population reporting no experience of vaginal intercourse was 40.0% (16–19 years), 10.9% (20–29 years) and 1.2% (30–39 years) for women and 35.0% (16–19 years), 9.6% (20–29 years) and 1.8% (30–39 years) for men [18]. The reasons for the substantially larger proportion of Japanese adults who report no heterosexual experience, as compared with those in other high-income countries, remain to be investigated.

Some of those who reported no heterosexual experience in our study most likely have experience from same-sex sexual activities. Studies from other countries show that between 2 and 5% of the population identify as having a sexual orientation other than heterosexual, [19, 20] although some of these individuals may have engaged in heterosexual intercourse and not all of them have same-sex sexual experience. Nonetheless, even if 5% of the young adults in Japan were to have engaged in same-sex sexual activities without ever having had heterosexual intercourse, around one in twenty 30-to-39-year-old women and men, according to our findings, would still lack sexual experience.

While positive sexual experiences are important contributors to quality of life, [9–12] some individuals may not consider the absence of sex as a source of dissatisfaction. In the Natsal-3 study in the UK, a substantial proportion of those with previous experience of sexual activity who had been sexually inactive in the year preceding survey participation did not report dissatisfaction with their (lack of) sex life [23]. Similarly, in a US study, those who had been sexually inactive for a year reported similar levels of happiness as their sexually active counterparts [16]. In Japan, it has been reported that some adults do not consider intimate relationships as being of high priority in their lives, with financial insecurity and long working hours possibly contributing to the purported trend [3, 24, 25]. The choice of not pursuing intimate relationships is gaining attention and acceptance, being described using terms such as “*soshokukei-danshi*”(herbivore men) and “*sekkusu-banare*” (drifting away from sex) [3, 24, 25]. However, difficulties in finding sexual partners may also explain sexual inexperience. In fact, around 80% of women and men aged 25–39 years who reported no heterosexual experience in our study responded that they wished to get married in their lifetime, indicating that their lack of sexual experience may be involuntary. The associations between several socioeconomic/regional variables and sexual inexperience in our study further supported this notion. Importantly, while the negative association of low income and unemployment with sexual inexperience among women aged 25–39 years could be explained by the relatively high proportion of married women who are housewives, [26] lower income as well as unstable or no employment were strongly associated with sexual inexperience among men. Although low income and the lack of sexual opportunity may share common determinants, these findings are in line with studies showing that high [27, 28] and stable [27] incomes are important predictors of attractiveness in the mating market, especially for men [8, 29]. Indeed, income is associated with marriage among Japanese men, [30] and it has been suggested that the decrease in stable employment opportunities over recent decades has contributed to low marriage and fertility rates in Japan [7, 31]. Japan’s low fertility rates persist despite the government’s efforts to encourage marriage, pregnancy and child rearing, including matchmaking events, fertility education programs and policies to improve work-life balance and childcare [2]. The situation of individuals who remain sexually inexperienced due to difficulties in finding a partner, as highlighted by our findings, may be considered in future policies aimed at increasing Japan’s birth rate.

Our study has limitations. First, as our analyses relied on self-reported data, results might be affected by under- or over-reporting of heterosexual inexperience due to social desirability bias; [32] however, the risk of such a bias may have been mitigated by the survey’s use of self-administered questionnaires [33]. Second, although the response rate in the National Fertility Survey was high (70.0 to 83.8% among unmarried persons and 85.7 to 92.5% among married couples) [8] and the sample was weighted to ensure that it was broadly representative of the Japanese population with respect to sex, age and marital status, non-response may have introduced bias into our findings. Third, data on stratification and sampling units used in the surveys were not available. While this did not affect the point estimates, [34, 35] the standard errors of the estimated prevalence and odds ratios could potentially have been influenced: the lack of stratification data may have led to an overestimation of the standard error, and the omission of sampling units may have underestimated the standard error [34, 35]. Confidence intervals in our analyses should therefore be interpreted with caution. Fourth, although the term *seikosho* used in the question regarding sexual experience is commonly used to refer to vaginal intercourse, the definition of this term was not specified in the survey and may have been interpreted differently by some participants. Fifth, our analyses underestimate the level of sexual inactivity in the population as those who have previous sexual experience may still be sexually inactive. Sexual inactivity may be more informative to assess than sexual inexperience with respect to the implications for fertility and public health. Sixth, as individual-level data were not available for the 2015 survey, we assessed factors associated with heterosexual inexperience in the 2010 survey. Seventh, as the question regarding sexual inexperience was only asked to unmarried individuals, we assumed that those who were or had been married had heterosexual experience; this may have led to an underestimation of sexual inexperience. Finally, as the information provided in the National Fertility Survey was limited to heterosexual experience, we could not assess same-sex sexual experience. There is a dearth of data on gay, lesbian, bisexual and transgender groups in Japan, [36] and questions targeted to the experiences of these groups should be included in future surveys.

## Conclusions

In this study using nationally representative survey data from Japan, we found that the proportion of men and women who reported no experience of heterosexual intercourse had increased in the past two decades. Around one in ten women and men remained heterosexually inexperienced into their thirties. Among men, temporary or part-time employment, unemployment and low income were associated with a higher likelihood of having no heterosexual experience. Further research is needed on the factors contributing to and the potential public health and demographic implications of the high proportion of young adults in Japan who remain sexually inexperienced well into adult age.

## Additional file

Additional file 1: Additional methods and 13 supplemental tables. (DOCX 127 kb)

## Abbreviations

aOR: age-adjusted odds ratio
IPSS: National Institute of Population and Social Security Research
JPY: Japanese Yen
