# Supplementary material for: Trends in heterosexual inexperience among young adults in Japan: analysis of national surveys, 1987–2015
Source: BMC Public Health. 2019 Apr 8;19:355. doi: 10.1186/s12889-019-6677-5 (PMC6452514; doi:10.1186/s12889-019-6677-5)
Supplement: Supplementary file 1 — Additional methods and 13 supplemental tables. (DOCX 127 kb) [file 12889_2019_6677_MOESM1_ESM.docx]

**Supplementary Material**

Ghaznavi C, Sakamoto H, Yoneoka D, Nomura S, Shibuya K, Ueda P*. Trends in heterosexual inexperience among young adults in Japan: analysis of national surveys, 1987-2015*

**The National Fertility Survey**

We used data from rounds 9 to 15 of the National Fertility Survey, conducted in 1987, 1992, 1997, 2002, 2005, 2010, and 2015. The survey was carried out by the National Institute of Population and Social Security Research, under the Japanese Ministry of Health, Labour, and Welfare to collect nationally representative data on topics related to marriage, child birth, and child rearing in Japan. Detailed information about the survey has been presented elsewhere.^1–10^ In brief, each survey used stratified cluster sampling with districts in the Population Census of Japan as primary sampling units, and comprised two national sub-surveys: one for married couples in which the wife was under 50 years of age, and one for unmarried women and men between 18 and 49 years of age, with the exception of the 1987 survey of unmarried adults which only included those aged 18-34 years.

Through home visits, participants were provided with a self-administered questionnaire which was returned upon completion in a sealed envelope at a follow-up visit. The married women provided information about their husbands; husband and wife were treated as two separate survey participants in our analyses, which were performed separately for each sex. The response rate across survey years ranged between 70.0% and 83.8% among unmarried individuals and between 85.7% and 92.5% among the married couples.^1^ We did not have information about the characteristics of the individuals who declined participation, and the Ministry of Health, Labour and Welfare had not used a pooled dataset with both sub-surveys in any of their previous analyses^1–10^ or provided sample weights to account for non-response. Therefore, we calculated sample weights based on sex, age and marital status using the Population Census of Japan, as described below, to ensure that the analyses produced nationally representative estimates.

Use of data on primary sampling units and stratum was not approved by the National Institute of Population and Social Security Research due to constraints on secondary data usage. We performed the analyses without accounting for stratification or sampling units. While this did not affect the point estimates,^11,12^ the standard errors of the estimated prevalence and odds ratios might have been affected; the lack of stratification data could have led to an overestimation of standard error and the omission of sampling units could have underestimated the standard error.^11,12^ Confidence intervals in our analyses should therefore be interpreted with caution.

**Calculation of sample weights**

Individual-level data were available for the National Fertility Surveys of 1987, 1992, 1997, 2002, 2005 and 2010. In these surveys, we used sample weights to adjust for potentially different rates of non-response and unknown status of heterosexual experience by age, sex and marital status (married/unmarried). Sample weights, which were defined as the inverse of the probability of being sampled according to age (1-year increments) and marital status in the Population Census of Japan, were calculated separately for men and women by using population weights and survey weights. Due to low numbers, married individuals between the ages 18 and 22 years were considered as one group in the calculation of sample weights.

*Age-standardized sample weights*

For each sex, the population weight for any one age and marital status group was calculated as the proportion of the total number of adults (of the same sex), aged 18-39 years, in the census who belonged to that group. Population weights were standardized to the age distribution of 18-39 year-old adults, by sex, in the 2015 Population Census as follows:

*Population weight = Proportion of the age group within the marital status category (for a given year) * proportion of total n of adults aged 18-39 years within the age group in 2015.*

To calculate these weights, we used Population Census data on the number of married and unmarried individuals in each age-sex-group from 1985, 1990, 1995, 2000, 2005, and 2010. For the survey years of 1987, 1992, 1997 and 2002, we estimated the proportion of married individuals in each age-sex group by log-linear interpolation of the proportion married taken from the censuses conducted before and after the survey year; the proportion of unmarried individuals was calculated as the married proportion subtracted from one. For example, to estimate the proportion of 35-year-old women who were married in 2002, we performed a log-linear interpolation using data on the proportion of married 35-year-old women taken from the 2000 and 2005 censuses. For the 2005 and 2010 surveys, we used data from the census conducted in the same year.

Survey weights were calculated as the proportion of the total number of 18-39-year-old survey participants, by sex, within the age and marital status group, after exclusion of participants with unknown status of sexual inexperience.

The age-standardized sample weights for each combination of age and marital status category were calculated for each sex and survey by dividing the population weight by the survey weight.

*Sample weights for 2010 without age-standardization*

For the 2010 survey, we calculated another set of sample weights, without age-standardization, which were used for the extrapolation of population characteristics in the 2010 survey to the Japanese population in 2010 and the logistic regression analyses in which the association of socioeconomic and regional factors with heterosexual inexperience was assessed. The sample weights were calculated using population weights that were based on the age distribution of 2010. After application of the sample weights in the 2010 survey, the distributions of age and marital status by sex closely matched those of the 2010 Population Census of Japan. (Supplemental Table 5)

**Estimation of prevalence of heterosexual inexperience in 2015**

The National Institute of Population and Social Security Research did not provide access to individual level data from the 2015 National Fertility Survey for this study. Therefore, we used summary data from in the institute’s official report of the survey^1^ and data from the 2015 Population Census of Japan to estimate the prevalence of heterosexual inexperience in 2015.

From the report, we obtained information about the proportion of never-married survey participants who reported that they had never had sexual intercourse with someone of the opposite sex by age group and sex. We multiplied these proportions with the proportion of never-married individuals in the country, estimated from the Population Census of Japan, in the corresponding age-sex-group. (Supplemental Table 6). Confidence intervals were not calculated.

In accordance with the use of sample weights in the surveys with individual-level data, the use of data from the Population Census of Japan meant that the prevalence estimates for 2015 accounted for potential differences in non-response by sex, age and marital status and were nationally representative with respect to these variables. Nonetheless, to ensure comparability with results from the surveys of 1987-2010, we compared the estimates obtained through the method of using the summary data from the 2010 report and the Population Census of Japan to the use of individual level data from the 2010 survey and sample weights (without age-standardization). The point estimates for prevalence obtained through the two methods were largely similar. (Supplemental Table 7)

**Supplemental Table 1** Number and characteristics of unmarried women in the National Fertility Survey, 1987-2010, who were excluded due to unknown heterosexual experience status vs. those who were included in the analyses. Numbers are shown in n(%).

| **Survey year** | **1987** | | **1992** | | **1997** | | **2002** | | **2005** | | **2010** | |
| --- | --- | --- | --- | --- | --- | --- | --- | --- | --- | --- | --- | --- |
|  | **Included** | **Excluded** | **Included** | **Excluded** | **Included** | **Excluded** | **Included** | **Excluded** | **Included** | **Excluded** | **Included** | **Excluded** |
| **n (%)** | 2561 (95) | 129 (5) | 3753 (94) | 240 (6) | 3758 (94) | 254 (6) | 3733 (91) | 353 (9) | 3247 (88) | 456 (12) | 3923 (93) | 313 (7) |
| **Age** |  |  |  |  |  |  |  |  |  |  |  |  |
| 18-19 | 600 (23) | 11 (9) | 831 (22) | 20 (8) | 563 (15) | 23 (9) | 542 (15) | 33 (9) | 498 (15) | 30 (7) | 492 (13) | 41 (13) |
| 20-24 | 1326 (52) | 57 (44) | 1724 (46) | 101 (42) | 1673 (45) | 100 (39) | 1329 (36) | 118 (33) | 1114 (34) | 137 (30) | 1323 (34) | 82 (26) |
| 25-29 | 470 (18) | 32 (25) | 726 (19) | 75 (31) | 909 (24) | 73 (29) | 980 (26) | 103 (29) | 772 (24) | 123 (27) | 899 (23) | 79 (25) |
| 30-34 | 165 (6) | 29 (22) | 280 (7) | 28 (12) | 389 (10) | 37 (15) | 555 (15) | 72 (20) | 528 (16) | 105 (23) | 663 (17) | 60 (19) |
| 35-39 |  |  | 192 (5) | 16 (7) | 224 (6) | 21 (8) | 327 (9) | 27 (8) | 335 (10) | 61 (13) | 546 (14) | 51 (16) |
| **Education^*^** |  |  |  |  |  |  |  |  |  |  |  |  |
| High school or less | 1196 (47) | 68 (53) | 1751 (47) | 121 (50) | 1406 (37) | 88 (35) | 1398 (37) | 118 (33) | 1099 (34) | 127 (28) | 1314 (33) | 84 (27) |
| Vocational school or short college | 961 (38) | 45 (35) | 1383 (37) | 71 (30) | 1507 (40) | 100 (39) | 1420 (38) | 152 (43) | 1152 (35) | 189 (41) | 1315 (34) | 128 (41) |
| Undergraduate or graduate studies | 384 (15) | 13 (10) | 610 (16) | 41 (17) | 812 (22) | 59 (23) | 861 (23) | 64 (18) | 954 (29) | 122 (27) | 1246 (32) | 87 (28) |
| Other/missing | 20 (1) | 3 (2) | 9 (0) | 7 (3) | 33 (1) | 7 (3) | 54 (1) | 19 (5) | 42 (1) | 18 (4) | 48 (1) | 14 (4) |
| **Occupational Status** |  |  |  |  |  |  |  |  |  |  |  |  |
| Regular employee | 1670 (65) | 96 (74) | 2490 (66) | 167 (70) | 2030 (54) | 141 (56) | 1626 (44) | 165 (47) | 1286 (40) | 192 (42) | 1593 (41) | 119 (38) |
| Part-time or temporary worker | 107 (4) | 4 (3) | 183 (5) | 10 (4) | 566 (15) | 42 (17) | 844 (23) | 68 (19) | 860 (26) | 116 (25) | 995 (25) | 80 (26) |
| Business owner or family business | 57 (2) | 10 (8) | 50 (1) | 9 (4) | 110 (3) | 10 (4) | 119 (3) | 9 (3) | 72 (2) | 12 (3) | 94 (2) | 5 (2) |
| Unemployed | 186 (7) | 8 (6) | 209 (6) | 16 (7) | 230 (6) | 18 (7) | 324 (9) | 26 (7) | 237 (7) | 36 (8) | 349 (9) | 25 (8) |
| Student | 511 (20) | 7 (5) | 778 (21) | 20 (8) | 707 (19) | 34 (13) | 632 (17) | 30 (8) | 718 (22) | 53 (12) | 744 (19) | 49 (16) |
| Other/missing | 30 (1) | 4 (3) | 43 (1) | 18 (8) | 115 (3) | 9 (4) | 188 (5) | 55 (16) | 74 (2) | 47 (10) | 148 (4) | 35 (11) |
| **Region of Residence** |  |  |  |  |  |  |  |  |  |  |  |  |
| Kanto | 108 (4) | 8 (6) | 112 (3) | 8 (3) | 151 (4) | 14 (6) | 148 (4) | 11 (3) | 130 (4) | 14 (3) | 122 (3) | 10 (3) |
| Hokkaido | 145 (6) | 8 (6) | 250 (7) | 12 (5) | 180 (5) | 14 (6) | 300 (8) | 12 (3) | 245 (8) | 13 (3) | 287 (7) | 19 (6) |
| Tohoku | 831 (32) | 51 (40) | 1302 (35) | 78 (32) | 1296 (34) | 76 (30) | 1189 (32) | 134 (38) | 1084 (33) | 165 (36) | 1318 (34) | 119 (38) |
| Chubu | 512 (20) | 15 (12) | 715 (19) | 50 (21) | 804 (21) | 54 (21) | 675 (18) | 73 (21) | 566 (17) | 81 (18) | 768 (20) | 57 (18) |
| Kinki | 417 (16) | 22 (17) | 641 (17) | 45 (19) | 561 (15) | 46 (18) | 655 (18) | 60 (17) | 567 (17) | 70 (15) | 637 (16) | 51 (16) |
| Chugoku/Shikoku | 201 (8) | 10 (8) | 304 (8) | 21 (9) | 377 (10) | 28 (11) | 320 (9) | 18 (5) | 221 (7) | 39 (9) | 317 (8) | 18 (6) |
| Kyushu/Okinawa | 347 (14) | 15 (12) | 429 (11) | 26 (11) | 389 (10) | 22 (9) | 446 (12) | 45 (13) | 434 (13) | 74 (16) | 474 (12) | 39 (12) |
| **Population Density of Residence (DiD) (in 10,000s)** |  |  |  |  |  |  |  |  |  |  |  |  |
| non-DiD | 832 (32) | 44 (34) | 1223 (33) | 75 (31) | 1028 (27) | 64 (25) | 1201 (32) | 97 (27) | 1183 (36) | 154 (34) | 1306 (33) | 105 (34) |
| < 20 | 685 (27) | 32 (25) | 964 (26) | 65 (27) | 1023 (27) | 72 (28) | 926 (25) | 103 (29) | 762 (23) | 114 (25) | 817 (21) | 71 (23) |
| 20-100 | 559 (22) | 23 (18) | 777 (21) | 51 (21) | 914 (24) | 63 (25) | 796 (21) | 76 (22) | 656 (20) | 97 (21) | 979 (25) | 77 (25) |
| ≥100 | 485 (19) | 30 (23) | 789 (21) | 49 (20) | 793 (21) | 55 (22) | 810 (22) | 77 (22) | 646 (20) | 91 (20) | 821 (21) | 60 (19) |

When including married women in the National Fertility Survey, the proportion of women with unknown heterosexual experience status was 2% (129/5995) in 1987, 3% (240/9158) in 1992, 3% (254/8017) in 1997, 4% (353/8234) in 2002, 7% (456/6983) in 2005, and 4% (313/7999) in 2010.

**Supplemental Table 2** Number and characteristics of unmarried men in the National Fertility Survey, 1987-2010, who were excluded due to unknown heterosexual experience status vs. those who were included in the analyses. Numbers are shown in n(%).

| **Survey year** | **1987** | | **1992** | | **1997** | | **2002** | | **2005** | | **2010** | |
| --- | --- | --- | --- | --- | --- | --- | --- | --- | --- | --- | --- | --- |
|  | **Included** | **Excluded** | **Included** | **Excluded** | **Included** | **Excluded** | **Included** | **Excluded** | **Included** | **Excluded** | **Included** | **Excluded** |
| **n (%)** | 3226 (96) | 149 (4) | 4490 (96) | 206 (4) | 4212 (96) | 194 (4) | 4175 (93) | 313 (7) | 3413 (89) | 438 (11) | 4267 (95) | 230 (5) |
| **Age** |  |  |  |  |  |  |  |  |  |  |  |  |
| 18-19 | 560 (17) | 25 (17) | 789 (18) | 37 (18) | 577 (14) | 21 (11) | 662 (16) | 38 (12) | 382 (11) | 54 (12) | 392 (9) | 32 (14) |
| 20-24 | 1416 (44) | 70 (47) | 1815 (40) | 72 (35) | 1619 (38) | 78 (40) | 1337 (32) | 103 (33) | 964 (28) | 112 (26) | 1328 (31) | 68 (30) |
| 25-29 | 839 (26) | 33 (22) | 1024 (23) | 47 (23) | 1137 (27) | 54 (28) | 1107 (27) | 88 (28) | 954 (28) | 128 (29) | 1072 (25) | 52 (23) |
| 30-34 | 411 (13) | 21 (14) | 503 (11) | 34 (17) | 554 (13) | 29 (15) | 697 (17) | 55 (18) | 653 (19) | 91 (21) | 819 (19) | 40 (17) |
| 35-39 | - | - | 359 (8) | 16 (8) | 325 (8) | 12 (6) | 372 (9) | 29 (9) | 460 (13) | 53 (12) | 656 (15) | 38 (17) |
| **Education^*^** |  |  |  |  |  |  |  |  |  |  |  |  |
| High school or less | 1660 (51) | 92 (62) | 2056 (46) | 97 (47) | 1917 (46) | 88 (45) | 1816 (43) | 140 (45) | 1418 (42) | 205 (47) | 1800 (42) | 95 (41) |
| Vocational school or short college | 386 (12) | 24 (16) | 622 (14) | 24 (12) | 698 (17) | 42 (22) | 836 (20) | 56 (18) | 571 (17) | 55 (13) | 697 (16) | 43 (19) |
| Undergraduate or graduate studies | 1134 (35) | 27 (18) | 1795 (40) | 67 (33) | 1548 (37) | 57 (29) | 1442 (35) | 98 (31) | 1374 (40) | 149 (34) | 1721 (40) | 71 (31) |
| Other/missing | 46 (1) | 6 (4) | 17 (0) | 18 (9) | 49 (1) | 7 (4) | 81 (2) | 19 (6) | 50 (1) | 29 (7) | 49 (1) | 21 (9) |
| **Occupational Status** |  |  |  |  |  |  |  |  |  |  |  |  |
| Regular employee | 2022 (63) | 92 (62) | 2849 (63) | 129 (63) | 2487 (59) | 110 (57) | 1976 (47) | 114 (36) | 1709 (50) | 181 (41) | 2015 (47) | 95 (41) |
| Part-time or temporary worker | 71 (2) | 0 (0) | 94 (2) | 4 (2) | 328 (8) | 15 (8) | 502 (12) | 28 (9) | 542 (16) | 74 (17) | 638 (15) | 25 (11) |
| Business owner or family business | 234 (7) | 15 (10) | 191 (4) | 11 (5) | 255 (6) | 20 (10) | 266 (6) | 14 (4) | 232 (7) | 24 (5) | 233 (5) | 12 (5) |
| Unemployed | 88 (3) | 5 (3) | 111 (2) | 2 (1) | 140 (3) | 5 (3) | 295 (7) | 25 (8) | 231 (7) | 20 (5) | 388 (9) | 12 (5) |
| Student | 761 (24) | 28 (19) | 1183 (26) | 39 (19) | 822 (20) | 25 (13) | 878 (21) | 49 (16) | 620 (18) | 62 (14) | 791 (19) | 40 (17) |
| Other/missing | 50 (2) | 9 (6) | 62 (1) | 21 (10) | 180 (4) | 19 (10) | 258 (6) | 83 (27) | 79 (2) | 77 (18) | 202 (5) | 46 (20) |
| **Region of Residence** |  |  |  |  |  |  |  |  |  |  |  |  |
| Kanto | 75 (2) | 4 (3) | 153 (3) | 4 (2) | 155 (4) | 3 (2) | 143 (3) | 10 (3) | 119 (3) | 16 (4) | 125 (3) | 8 (3) |
| Hokkaido | 261 (8) | 12 (8) | 483 (11) | 12 (6) | 214 (5) | 8 (4) | 322 (8) | 22 (7) | 236 (7) | 33 (8) | 327 (8) | 16 (7) |
| Tohoku | 1157 (36) | 60 (40) | 1580 (35) | 70 (34) | 1468 (35) | 79 (41) | 1485 (36) | 93 (30) | 1299 (38) | 126 (29) | 1423 (33) | 88 (38) |
| Chubu | 589 (18) | 18 (12) | 957 (21) | 42 (20) | 983 (23) | 48 (25) | 808 (19) | 78 (25) | 653 (19) | 93 (21) | 824 (19) | 39 (17) |
| Kinki | 527 (16) | 23 (15) | 503 (11) | 38 (18) | 496 (12) | 24 (12) | 625 (15) | 54 (17) | 501 (15) | 67 (15) | 645 (15) | 39 (17) |
| Chugoku/Shikoku | 260 (8) | 20 (13) | 369 (8) | 22 (11) | 442 (10) | 13 (7) | 394 (9) | 28 (9) | 232 (7) | 28 (6) | 384 (9) | 19 (8) |
| Kyushu/Okinawa | 357 (11) | 12 (8) | 445 (10) | 18 (9) | 454 (11) | 19 (10) | 398 (10) | 28 (9) | 373 (11) | 75 (17) | 539 (13) | 21 (9) |
| **Population Density and Size of Residence (in 10,000s)** |  |  |  |  |  |  |  |  |  |  |  |  |
| Non-densely inhabited area | 1137 (35) | 62 (42) | 1478 (33) | 89 (43) | 1243 (30) | 60 (31) | 1419 (34) | 101 (32) | 1238 (36) | 192 (44) | 1434 (34) | 77 (33) |
| < 20 | 746 (23) | 35 (23) | 1336 (30) | 58 (28) | 1074 (25) | 49 (25) | 935 (22) | 70 (22) | 720 (21) | 82 (19) | 937 (22) | 56 (24) |
| 20-100 | 615 (19) | 27 (18) | 864 (19) | 33 (16) | 1079 (26) | 46 (24) | 892 (21) | 81 (26) | 799 (23) | 82 (19) | 1070 (25) | 49 (21) |
| ≥100 | 728 (23) | 25 (17) | 812 (18) | 26 (13) | 816 (19) | 39 (20) | 929 (22) | 61 (19) | 656 (19) | 82 (19) | 826 (19) | 48 (21) |

When including married men in the National Fertility Survey, the proportion of men with unknown heterosexual experience status was 3% (149/5558) in 1987, 2% (206/8692) in 1992, 3% (194/7480) in 1997, 4% (313/7753) in 2002, 7% (438/6402) in 2005, and 3% (230/7503) in 2010.

**Supplemental Table 3** Number of men and women who were included in the analyses from each round of the National Fertility Survey 1987-2015.

|  | Women | Men |
| --- | --- | --- |
| Survey Year | n (n unmarried) | n (n unmarried) |
| 1987 | 5,995 (2,561) | 5,558 (3,226) |
| 1992 | 9,158 (3,753) | 8,692 (4,490) |
| 1997 | 8,017 (3,758) | 7,480 (4,212) |
| 2002 | 8,234 (3,733) | 7,753 (4,175) |
| 2005 | 6,983 (3,247) | 6,402 (3,413) |
| 2010 | 7,999 (3,923) | 7,503 (4,267) |

**Supplemental Table 4** Questions regarding heterosexual inexperience in the National Fertility Survey, 1987-2015.

| Survey Year | Question | Answers |
| --- | --- | --- |
| 1987 | Have you ever had sexual intercourse with someone of the opposite sex? | Yes No |
| 1992 | Have you ever had sexual intercourse with someone of the opposite sex? | Yes No |
| 1997 | Have you ever had sexual intercourse with someone of the opposite sex? | Yes No |
| 2002 | Have you ever had sexual intercourse with someone of the opposite sex? | Yes  No |
| 2005 | Have you ever had (1) sexual intercourse with someone of the opposite sex? If you have, in the most previous occasion (2) did you use contraception, and (3) what method was that?^*^ | Yes, within the previous year.  Yes, not in the previous year but before that. No |
| 2010 | Have you ever had sexual intercourse with someone of the opposite sex? | Yes  No |
| 2015 | Have you ever had (1) sexual intercourse with someone of the opposite sex? If you have, in the most previous occasion (2) did you use contraception, and (3) what method was that?^*^ | Yes  No |

^*^ The questions regarding contraception were answered separately from the question about experience of sexual intercourse.

**Supplemental Table 5** Distribution of age and marital status by sex in the 2010 National Fertility Survey after application of sample weights, and in the 2010 Population Census of Japan. Numbers are shown in percent.

|  | Women | | | | Men | | | |
| --- | --- | --- | --- | --- | --- | --- | --- | --- |
|  | National Fertility Survey 2010 | | Population Census 2010 | | National Fertility Survey 2010 | | Population Census 2010 | |
| Age  group | Married | Unmarried | Married | Unmarried | Married | Unmarried | Married | Unmarried |
| 18-19 | <0.1 | 7.0 | <0.1 | 7.1 | <0.1 | 7.1 | <0.1 | 7.3 |
| 20-24 | 1.8 | 16.8 | 1.7 | 16.8 | 1.1 | 17.6 | 1.1 | 17.6 |
| 25-29 | 7.7 | 13.4 | 7.7 | 13.4 | 5.7 | 15.4 | 5.7 | 15.3 |
| 30-34 | 14.8 | 9.6 | 14.8 | 9.6 | 12.3 | 12.1 | 12.2 | 12.1 |
| 35-39 | 20.1 | 8.7 | 20.1 | 8.7 | 17.5 | 11.2 | 17.6 | 11.3 |
| Total | 44.4 | 55.5 | 44.4 | 55.6 | 36.6 | 63.4 | 36.6 | 63.5 |

**Supplemental Table 6** Data from the report of the National Fertility Survey 2015^1^ and the Population Census^13^ used to estimate prevalence of heterosexual inexperience in 2015.

|  | Women | | Men | | |
| --- | --- | --- | --- | --- | --- |
| Age group | % sexually inexperienced among never-married^*^ | % never married^†^ | % sexually inexperienced among never-married^*^ | % never married^†^ |  |
| 18-19 | 78.4 | 98.8 | 75.7 | 99.2 |  |
| 20-24 | 48.5 | 91.5 | 49.0 | 95.1 |  |
| 25-29 | 34.7 | 61.7 | 32.8 | 72.8 |  |
| 30-34 | 34.1 | 34.9 | 26.8 | 47.3 |  |
| 35-39 | 37.2 | 24.0 | 27.1 | 35.2 |  |

^*^ Excluding participants with unknown status of heterosexual experience. Estimates were calculated from the proportions reported in Table I-2-4 in the 2015 National Fertility Survey report.^1^ The reported proportions were based on responses from 2,893 women and 3,163 men, including women and men with unknown status of heterosexual experience. The proportions of women with unknown status of heterosexual experience were 5.0% (18-19 years), 4.2% (20-24 years), 6.1% (25-29 years), 8.3% (30-34 years), and 10.3% (35-39 years). The proportions of men with unknown status of heterosexual experience were 3.8% (18-19 years), 4.1% (20-24 years), 3.2% (25-29 years), 4.3% (30-34 years), and 4.1% (35-39 years).^1^

^†^ Data from the Population Census of Japan, 2015.

**Supplemental Table 7** Comparison of prevalence of heterosexual inexperience (%) in 2010 estimated using summary data from the report of the National Fertility Survey and the Population Census^13^ versus individual-level data and sample weights in the survey.

|  | Women | | Men | |
| --- | --- | --- | --- | --- |
| Age group | Summary data | Survey data | Summary data | Survey data |
| 18-19 | 69.8 | 71.4 | 71.9 | 72.5 |
| 20-24 | 37.9 | 37.7 | 39.4 | 39.7 |
| 25-29 | 19.2 | 19.1 | 18.6 | 18.3 |
| 30-34 | 9.0 | 8.7 | 12.9 | 12.7 |
| 35-39 | 6.6 | 6.1 | 10.2 | 10.2 |
| 18-39 | 20.1 | 19.9 | 22.5 | 22.5 |

**Supplemental Table 8** Definitions and categorization of socioeconomic and regional variables used for calculating age-adjusted odds ratios for heterosexual inexperience in the National Fertility Survey 2010.

| **Predictor** | **Categorization** |
| --- | --- |
| Age | Continuous variable |
| Education^*^ | High school or less; vocational school or short-term college; undergraduate studies; graduate studies. |
| Occupational status | Regular employee; part-time or temporary worker; business owner or member of family business; unemployed; student. |
| Annual income in 10,000 Japanese Yen (JPY) ^† ‡^ | *18-24 years*: 0-99; 100-299; 300 or more  *25-39 years*: 0-99; 100-299; 300-499; 500-799 (500 or more for women); 800 or more (men) |
| Region of residence^**^ | Kanto; Hokkaido; Tohoku; Chubu; Kinki; Chugoku/Shikoku; Kyushu/Okinawa |
| Population density and size of residence | Non-densely inhabited district; district with less than 200,000 inhabitants; between 200,000 and 1,000,000 inhabitants; more than 1,000,000 inhabitants^14^ |

^*^ Education was not included as a variable in the analysis of 18-24 year-old women and men because most adults in younger ages may not have concluded their studies. Currently enrolled students were categorized according to their ongoing education.

^†^ 10,000 JPY was approximately 78 Euro as of June 2018.

^‡^ Income was categorized according to the individual’s revenues.

^**^ The seven regions constitute geographically clustered prefectures (the highest administrative divisions of Japan) and are often used in discussion of regional economic and policy issues in the country.

|  |  | Age group | | | | | |
| --- | --- | --- | --- | --- | --- | --- | --- |
|  | Year | 18-39 | 18-19 | 20-24 | 25-29 | 30-34 | 35-39 |
| Total | 1987 | - | 80.7 (77.5 - 83.6) | 55.5 (53.1 - 57.9) | 19.6 (17.6 - 21.8) | 6.2 (4.9 - 7.8) | - |
|  | 1992 | 21.7 (20.8 - 22.6) | 78.1 (75.2 - 80.8) | 47.8 (45.7 - 50.0) | 20.7 (18.9 - 22.7) | 7.3 (6.0 - 8.7) | 4.0 (3.1 - 5.1) |
|  | 1997 | 18.8 (18.0 - 19.7) | 70.7 (66.9 - 74.3) | 39.4 (37.2 - 41.6) | 18.3 (16.6 - 20.2) | 6.6 (5.5 - 8.0) | 3.6 (2.7 - 4.7) |
|  | 2002 | 18.0 (17.1 - 18.9) | 65.1 (61.1 - 69.0) | 36.4 (34.0 - 38.9) | 15.9 (14.2 - 17.7) | 8.4 (7.2 - 9.9) | 4.2 (3.3 - 5.4) |
|  | 2005 | 18.6 (17.7 - 19.6) | 67.3 (63.1 - 71.3) | 36.0 (33.4 - 38.7) | 17.1 (15.1 - 19.3) | 9.8 (8.4 - 11.4) | 4.3 (3.3 - 5.5) |
|  | 2010 | 19.9 (19.0 - 20.9) | 71.4 (67.3 - 75.2) | 37.7 (35.3 - 40.3) | 19.1 (17.2 - 21.3) | 8.7 (7.4 - 10.1) | 6.1 (5.1 - 7.3) |
|  | 2015 | 24.6 | 77.5 | 44.4 | 21.4 | 11.9 | 8.9 |
|  | p (linear)* | 0.001 | 0.006 | < 0.001 | 0.069 | 0.789 | 0.012 |
|  | p (quadratic)* | 0.001 | 0.007 | < 0.001 | 0.061 | 0.398 | 0.003 |
| Never-married | 1987 | - | 82.8 (79.6 - 85.6) | 67.0 (64.4 - 69.5) | 57.8 (53.1 - 62.3) | 53.4 (44.7 - 61.9) | - |
|  | 1992 | 56.7 (55.0 - 58.4) | 79.3 (76.4 - 81.9) | 55.8 (53.4 - 58.2) | 48.9 (45.2 - 52.7) | 45.0 (38.6 - 51.4) | 45.7 (37.0 - 54.6) |
|  | 1997 | 45.2 (43.5 - 46.9) | 72.0 (68.2 - 75.6) | 45.1 (42.7 - 47.5) | 37.2 (34.0 - 40.5) | 31.6 (26.6 - 36.9) | 35.0 (27.4 - 43.6) |
|  | 2002 | 39.4 (37.7 - 41.0) | 66.6 (62.5 - 70.4) | 41.3 (38.7 - 44.0) | 28.1 (25.3 - 31.1) | 31.0 (26.9 - 35.5) | 31.5 (25.3 - 38.5) |
|  | 2005 | 39.2 (37.4 - 41.0) | 67.8 (63.5 - 71.8) | 40.7 (37.9 - 43.7) | 29.1 (25.9 - 32.5) | 33.8 (29.4 - 38.5) | 26.5 (20.9 - 32.9) |
|  | 2010 | 39.5 (37.9 - 41.1) | 71.7 (67.6 - 75.5) | 42.3 (39.7 - 45.0) | 32.0 (29.0 - 35.3) | 25.9 (22.4 - 29.7) | 28.5 (24.2 - 33.2) |
|  | 2015 | 46.0 | 78.4 | 48.5 | 34.7 | 34.1 | 37.2 |
|  | p (linear)* | < 0.001 | 0.003 | < 0.001 | 0.001 | 0.010 | 0.004 |
|  | p (quadratic)* | < 0.001 | 0.005 | < 0.001 | 0.003 | 0.026 | 0.006 |

**Supplemental Table 9** Prevalence of heterosexual inexperience among women, standardized to the age-distribution in 2015, in the total population and in those who have never been married, 1987-2015. Prevalence is shown in percent (95% CI). Raw data for figures 1 and 2.

^*^ Linear and quadratic trends in prevalence across the survey years were assessed using linear regression. P-values of less than 0.05 were regarded as statistically significant.

|  |  | Age group | | | | | |
| --- | --- | --- | --- | --- | --- | --- | --- |
|  |  | 18-39 | 18-19 | 20-24 | 25-29 | 30-34 | 35-39 |
| Total | 1987 | - | 74.9 (71.2 - 78.3) | 41.9 (39.5 - 44.4) | 18.9 (17.0 - 21.1) | 8.8 (7.4 - 10.5) | - |
|  | 1992 | 20.0 (19.1 - 20.8) | 74.4 (71.3 - 77.3) | 40.4 (38.2 - 42.6) | 16.7 (14.9 - 18.6) | 8.2 (6.9 - 9.8) | 5.5 (4.4 - 6.7) |
|  | 1997 | 19.3 (18.4 - 20.2) | 68.3 (64.4 – 72.0) | 35.2 (32.9 - 37.4) | 17.9 (16.1 - 19.8) | 9.5 (8.0 - 11.2) | 6.2 (4.9 - 7.7) |
|  | 2002 | 19.5 (18.6 - 20.4) | 66.1 (62.4 - 69.6) | 34.2 (31.8 - 36.7) | 18.7 (16.8 - 20.7) | 10.5 (9.0 - 12.1) | 6.6 (5.3 - 8.2) |
|  | 2005 | 20.7 (19.7 - 21.8) | 67.8 (62.9 - 72.3) | 34.6 (31.8 - 37.6) | 18.8 (16.8 – 21.0) | 12.5 (10.9 - 14.4) | 8.3 (6.9 - 9.9) |
|  | 2010 | 22.5 (21.5 - 23.5) | 72.5 (67.9 - 76.7) | 39.7 (37.2 - 42.3) | 18.3 (16.4 - 20.4) | 12.7 (11.2 - 14.4) | 10.2 (8.9 - 11.7) |
|  | 2015 | 25.8 | 75.1 | 46.6 | 23.9 | 12.7 | 9.5 |
|  | p (linear)* | 0.002 | 0.017 | 0.004 | 0.168 | 0.248 | 0.372 |
|  | p (quadratic)* | 0.001 | 0.016 | 0.003 | 0.066 | 0.991 | 0.891 |
| Never-married | 1987 | - | 75.4 (71.6 - 78.7) | 45.3 (42.7 - 48.0) | 30.9 (27.8 - 34.2) | 29.6 (25.2 - 34.4) | - |
|  | 1992 | 38.2 (36.7 - 39.7) | 75.0 (71.8 - 77.9) | 43.4 (41.1 - 45.7) | 25.6 (23.0 - 28.4) | 24.3 (20.7 - 28.4) | 27.1 (22.4 - 32.3) |
|  | 1997 | 35.4 (33.9 - 36.9) | 68.8 (64.9 - 72.4) | 37.8 (35.4 - 40.2) | 26.8 (24.2 - 29.5) | 24.7 (21.2 - 28.7) | 26.5 (21.6 - 32.1) |
|  | 2002 | 34.3 (32.8 - 35.8) | 66.8 (63.1 - 70.3) | 36.6 (34.1 - 39.3) | 27.0 (24.4 - 29.7) | 24.8 (21.6 - 28.3) | 25.3 (20.8 - 30.5) |
|  | 2005 | 35.1 (33.5 - 36.8) | 67.9 (63.1 - 72.4) | 37.3 (34.3 - 40.4) | 26.5 (23.8 - 29.5) | 27.4 (24.0 - 31.1) | 28.3 (24.0 - 33.1) |
|  | 2010 | 36.8 (35.3 - 38.3) | 72.9 (68.3 - 77.1) | 42.3 (39.7 - 45.0) | 25.8 (23.2 - 28.5) | 27.2 (24.2 - 30.4) | 29.0 (25.4 - 32.8) |
|  | 2015 | 41.2 | 75.7 | 49.0 | 32.8 | 26.8 | 27.1 |
|  | p (linear)* | < 0.001 | 0.016 | 0.003 | 0.049 | 0.202 | 0.932 |
|  | p (quadratic)* | < 0.001 | 0.016 | 0.003 | 0.038 | 0.196 | 0.965 |

**Supplemental Table 10** Prevalence of heterosexual inexperience among men, standardized to the age-distribution in 2015, in the total population and in those who have never been married, 1987-2015. Prevalence is shown in percent (95% CI). Raw data for figures 1 and 3.

^*^ Linear and quadratic trends in prevalence across the survey years were assessed using linear regression. P-values of less than 0.05 were regarded as statistically significant.

**Supplemental Table 11** Characteristics of adults between the ages of 18 and 39 years in the 2010 National Fertility Survey, by sex and status of heterosexual inexperience.

|  | **Women** (N=7,999) | | **Men** (N=7,503) | |
| --- | --- | --- | --- | --- |
|  | **Heterosexual experience** | | **Heterosexual experience** | |
|  | **No** (n=1,429 [17.9%]) | **Yes** (n=6,570 [82.1%]) | **No** (n=1,497 [20.0%]) | **Yes** (n=6,006 [80.0%]) |
|  | **n (%)** | **n (%)** | **n (%)** | **n (%)** |
| **Age** |  |  |  |  |
| 18-19 | 350 (24.5) | 144 (2.2) | 285 (19.0) | 110 (1.8) |
| 20-24 | 553 (38.7) | 911 (13.9) | 561 (37.5) | 849 (14.1) |
| 25-29 | 271 (19.0) | 1227 (18.7) | 270 (18.0) | 1267 (21.1) |
| 30-34 | 145 (10.2) | 1810 (27.6) | 210 (14.0) | 1660 (27.6) |
| 35-39 | 110 (7.7) | 2478 (37.7) | 171 (11.4) | 2120 (35.3) |
| **Marital Status** |  |  |  |  |
| Unmarried | 1429 (100) | 2494 (38.0) | 1497 (100) | 2770 (46.1) |
| Married | 0 (0) | 4076 (62.0) | 0 (0) | 3236 (53.9) |
| **Education^*^** |  |  |  |  |
| High school or less | 440 (31.3) | 2542 (39.0) | 627 (42.6) | 2647 (44.5) |
| Vocational school or short college | 422 (30.1) | 2490 (38.2) | 238 (16.2) | 1032 (17.3) |
| Undergraduate studies | 520 (37.0) | 1386 (21.3) | 544 (37.0) | 1962 (33.0) |
| Graduate studies | 22 (1.6) | 101 (1.5) | 62 (4.2) | 310 (5.2) |
| **Occupational Status** |  |  |  |  |
| Regular employee | 444 (32.6) | 1928 (30.4) | 474 (31.6) | 4106 (71.5) |
| Part-time or temporary worker | 323 (23.7) | 1882 (29.6) | 323 (21.5) | 560 (9.7) |
| Business owner or family business | 21 (1.5) | 251 (4.0) | 45 (3.0) | 502 (8.7) |
| Unemployed | 125 (9.2) | 1980 (31.2) | 201 (13.4) | 232 (4.0) |
| Student | 451 (33.1) | 307 (4.8) | 459 (30.6) | 344 (6.0) |
| **Annual Income (in JPY 10,000s)**^†^ |  |  |  |  |
| 0-99 | 820 (59.5) | 3640 (57.0) | 876 (60.8) | 1115 (19.2) |
| 100-299 | 414 (30.1) | 1774 (27.8) | 353 (24.5) | 1365 (23.5) |
| 300-499 | 130 (9.4) | 811 (12.7) | 185 (12.8) | 2242 (38.7) |
| 500-799^‡^ | 13 (0.9) | 157 (2.5) | 25 (1.7) | 927 (16.0) |
| ≥800 | - | - | 2 (0.1) | 150 (2.6) |
| **Region of Residence** |  |  |  |  |
| Kanto | 494 (34.6) | 2171 (33.0) | 531 (35.5) | 1963 (32.7) |
| Hokkaido | 44 (3.1) | 233 (3.6) | 31 (2.1) | 213 (3.6) |
| Tohoku | 94 (6.6) | 514 (7.8) | 118 (7.9) | 474 (7.9) |
| Chubu | 278 (19.5) | 1337 (20.4) | 307 (20.5) | 1201 (20.0) |
| Kinki | 261 (18.3) | 980 (14.9) | 241 (16.1) | 867 (14.4) |
| Chugoku/Shikoku | 119 (8.3) | 556 (8.5) | 123 (8.2) | 546 (9.1) |
| Kyushu/Okinawa | 139 (9.7) | 779 (11.9) | 146 (9.8) | 742 (12.4) |
| **Population Density and Size of Residence (in 10,000s)** |  |  |  |  |
| Non-densely inhabited area | 506 (35.4) | 2197 (33.4) | 519 (34.7) | 2030 (33.8) |
| < 20 | 305 (21.3) | 1407 (21.4) | 326 (21.8) | 1331 (22.2) |
| 20-100 | 319 (22.3) | 1630 (24.8) | 367 (24.5) | 1445 (24.1) |
| ≥100 | 299 (20.9) | 1336 (20.3) | 285 (19.0) | 1200 (20.0) |
| **Wish to get married in lifetime** |  |  |  |  |
| No | 191 (13.9) | - | 244 (17.2) | - |
| Yes | 1183 (86.1) | - | 1174 (82.8) | - |

^*^ Currently enrolled students were categorized according to their ongoing education.

^†^ The survey included questions on annual income and income during the month preceding survey participation. For participants missing a response on annual income, income from the previous month was used to estimate the annual income. For participants who had missing data on income and whose occupational status was listed as unemployed, annual income was set to zero.

^‡^ ≥ JPY 500 for women; JPY= Japanese Yen

Missing values were education (women, n=76; men, n=81), occupational status (women, n=287; men, n=348), annual income (women, n=240; men, n=263), wish to get married in lifetime (women, n=55; men, n=79).

**Supplemental Table 12** Characteristics of adults between the ages of 18 and 24 years in the 2010 National Fertility Survey, and extrapolation to all Japanese adults between the ages of 18 and 24 years, by sex and status of heterosexual experience.

|  | **National Fertility Survey** | | | | **Extrapolation to all Japanese adults aged 18-24 years** | | | |
| --- | --- | --- | --- | --- | --- | --- | --- | --- |
|  | **Women** (N=1,958) | | **Men** (N=1,805) | | **Women** (n=4,178) | | **Men** (n=4,353) | |
|  | **Heterosexual experience** | | **Heterosexual experience** | | **Heterosexual experience** | | **Heterosexual experience** | |
|  | **No** | **Yes** | **No** | **Yes** | **No** | **Yes** | **No** | **Yes** |
| N (%) | 903 (46.1) | 1055 (53.9) | 846 (46.9) | 959 (53.1) | 1962 (47.0) | 2216 (53.0) | 2126 (48.8) | 2227 (51.2) |
| **Age** |  |  |  |  |  |  |  |  |
| 18-19 | 350 (38.8) | 144 (13.7) | 285 (33.7) | 110 (11.5) | 818 (41.7) | 328 (14.8) | 878 (41.3) | 332 (14.9) |
| 20-24 | 553 (61.2) | 911 (86.4) | 561 (66.3) | 849 (88.5) | 1144 (58.3) | 1888 (85.2) | 1248 (58.7) | 1895 (85.1) |
| **Marital Status** |  |  |  |  |  |  |  |  |
| Unmarried | 903 (100) | 912 (86.5) | 846 (100) | 874 (91.1) | 1962 (100) | 1919 (86.6) | 2126 (100) | 2040 (91.6) |
| Married | 0 (0) | 143 (13.6) | 0 (0) | 85 (8.9) | 0 (0) | 297 (13.4) | 0 (0) | 187 (8.4) |
| **Education^*^** |  |  |  |  |  |  |  |  |
| High school or less | 276 (30.6) | 397 (37.6) | 301 (35.6) | 401 (41.8) | 608 (31.0) | 838 (37.8) | 778 (36.6) | 935 (42.0) |
| Vocational school or short college | 240 (26.6) | 317 (30.1) | 130 (15.4) | 148 (15.4) | 514 (26.2) | 665 (30.0) | 321 (15.1) | 343 (15.4) |
| Undergraduate studies | 363 (40.2) | 320 (30.3) | 376 (44.4) | 363 (37.9) | 787 (40.1) | 669 (30.2) | 935 (44.0) | 840 (37.7) |
| Graduate studies | 5 (0.6) | 14 (1.3) | 24 (2.8) | 35 (3.7) | 10 (0.5) | 29 (1.3) | 53 (2.5) | 78 (3.5) |
| Missing | 19 (2.1) | 7 (0.7) | 15 (1.8) | 12 (1.3) | 43 (2.2) | 16 (0.7) | 38 (1.8) | 29 (1.3) |
| **Occupational Status** |  |  |  |  |  |  |  |  |
| Regular employee | 213 (23.6) | 382 (36.2) | 198 (23.4) | 426 (44.4) | 447 (22.8) | 793 (35.8) | 478 (22.5) | 969 (43.5) |
| Part-time or temporary worker | 156 (17.3) | 220 (20.9) | 80 (9.5) | 112 (11.7) | 330 (16.8) | 456 (20.6) | 185 (8.7) | 256 (11.5) |
| Business owner or family business | 5 (0.6) | 9 (0.9) | 12 (1.4) | 22 (2.3) | 10 (0.5) | 20 (0.9) | 28 (1.3) | 51 (2.3) |
| Unemployed | 50 (5.5) | 128 (12.1) | 74 (8.8) | 48 (5.0) | 106 (5.4) | 270 (12.2) | 181 (8.5) | 114 (5.1) |
| Student | 447 (49.5) | 284 (26.9) | 444 (52.5) | 313 (32.6) | 999 (50.9) | 607 (27.4) | 1161 (54.6) | 746 (33.5) |
| Missing | 32 (3.5) | 32 (3.0) | 38 (4.5) | 38 (4.0) | 71 (3.6) | 69 (3.1) | 94 (4.4) | 91 (4.1) |
| **Annual Income (in JPY 10,000s)**^†^ |  |  |  |  |  |  |  |  |
| 0-99 | 654 (72.4) | 594 (56.3) | 640 (75.7) | 512 (53.4) | 1517 (77.3) | 1208 (54.5) | 1563 (73.5) | 1267 (56.9) |
| 100-299 | 194 (21.5) | 360 (34.1) | 141 (16.7) | 281 (29.3) | 308 (15.7) | 634 (28.6) | 438 (20.6) | 750 (33.7) |
| ≥300 | 31 (3.4) | 65 (6.2) | 37 (4.4) | 143 (14.9) | 78 (4.0) | 321 (14.5) | 70 (3.3) | 134 (6.0) |
| Missing | 24 (2.7) | 36 (3.4) | 28 (3.3) | 23 (2.4) | 59 (3.0) | 53 (2.4) | 57 (2.7) | 76 (3.4) |
| **Region of Residence** |  |  |  |  |  |  |  |  |
| Kanto | 320 (30.3) | 310 (34.3) | 275 (28.7) | 291 (34.4) | 673 (34.3) | 669 (30.2) | 733 (34.5) | 632 (28.4) |
| Chubu | 244 (23.1) | 175 (19.4) | 191 (19.9) | 186 (22.0) | 379 (19.3) | 512 (23.1) | 466 (21.9) | 441 (19.8) |
| Kinki | 161 (15.3) | 159 (17.6) | 159 (16.6) | 126 (14.9) | 345 (17.6) | 343 (15.5) | 317 (14.9) | 374 (16.8) |
| Kyushu/Okinawa | 137 (13.0) | 93 (10.3) | 136 (14.2) | 89 (10.5) | 202 (10.3) | 286 (12.9) | 230 (10.8) | 318 (14.3) |
| Chugoku/Shikoku | 75 (7.1) | 76 (8.4) | 88 (9.2) | 71 (8.4) | 167 (8.5) | 157 (7.1) | 174 (8.2) | 205 (9.2) |
| Tohoku | 83 (7.9) | 59 (6.5) | 79 (8.2) | 62 (7.3) | 129 (6.6) | 173 (7.8) | 153 (7.2) | 187 (8.4) |
| Hokkaido | 35 (3.3) | 31 (3.4) | 31 (3.2) | 21 (2.5) | 67 (3.4) | 73 (3.3) | 55 (2.6) | 71 (3.2) |
| **Population Density and Size of Residence(in 10,000s)** |  |  |  |  |  |  |  |  |
| Non-densely inhabited area | 317 (35.1) | 352 (33.4) | 282 (33.3) | 325 (33.9) | 685 (34.9) | 740 (33.4) | 702 (33.0) | 762 (34.2) |
| <20 | 192 (21.3) | 221 (21.0) | 185 (21.9) | 205 (21.4) | 418 (21.3) | 459 (20.7) | 470 (22.1) | 477 (21.4) |
| 20-100 | 184 (20.4) | 251 (23.8) | 195 (23.1) | 232 (24.2) | 402 (20.5) | 527 (23.8) | 493 (23.2) | 530 (23.8) |
| ≥100 | 210 (23.3) | 231 (21.9) | 184 (21.8) | 197 (20.5) | 457 (23.3) | 490 (22.1) | 461 (21.7) | 459 (20.6) |
| **Wish to get married in lifetime** |  |  |  |  |  |  |  |  |
| No | 97 (10.7) | - | 112 (13.2) | - | 212 (10.8) |  | 279 (13.1) |  |
| Yes | 781 (86.5) | - | 698 (82.5) | - | 1695 (86.4) |  | 1758 (82.7) |  |
| Missing | 25 (2.8) | - | 36 (4.3) | - | 55 (2.8) |  | 91 (4.3) |  |

Extrapolated numbers are shown in 1000s.

^*^ Currently enrolled students were categorized according to their ongoing education.

^†^ ≥ JPY 500 for women; JPY= Japanese Yen

**Supplemental Table 13** Characteristics of adults between the ages of 25 and 39 years in the 2010 National Fertility Survey, and extrapolation to all Japanese adults between the ages of 25 and 39 years, by sex and status of heterosexual experience.

|  | **National Fertility Survey** | | | | **Extrapolation to all Japanese adults aged 25-39 years** | | | |
| --- | --- | --- | --- | --- | --- | --- | --- | --- |
|  | **Women** (n=6041) | | **Men** (n=5698) | | **Women** (n=12152) | | **Men** (n=12507) | |
|  | **Heterosexual experience** | | **Heterosexual experience** | | **Heterosexual experience** | | **Heterosexual experience** | |
|  | **No** | **Yes** | **No** | **Yes** | **No** | **Yes** | **No** | **Yes** |
| N (%) | 526 (8.7) | 5515 (91.3) | 651 (11.4) | 5047 (88.6) | 1294 (10.6) | 10858 (89.4) | 1669 (13.3) | 10838 (86.7) |
| **Age** |  |  |  |  |  |  |  |  |
| 25-29 | 271 (51.5) | 1227 (22.3) | 270 (41.5) | 1267 (25.1) | 663 (51.2) | 2791 (25.7) | 651 (39.0) | 2905 (26.8) |
| 30-34 | 145 (27.6) | 1810 (32.8) | 210 (32.3) | 1660 (32.9) | 345 (26.7) | 3637 (33.5) | 522 (31.3) | 3587 (33.1) |
| 35-39 | 110 (20.9) | 2478 (44.9) | 171 (26.3) | 2120 (42.0) | 287 (22.2) | 4419 (40.7) | 494 (29.6) | 4357 (40.2) |
| **Marital Status** |  |  |  |  |  |  |  |  |
| Unmarried | 526 (100) | 1582 (28.7) | 651 (100) | 1896 (37.6) | 1294 (100) | 3898 (35.9) | 1669 (100) | 4855 (44.8) |
| Married | 0 (0) | 3933 (71.3) | 0 (0) | 3151 (62.4) | 0 (0) | 6960 (64.1) | 0 (0) | 5983 (55.2) |
| **Education^*^** |  |  |  |  |  |  |  |  |
| High school or less | 164 (31.2) | 2145 (38.9) | 326 (50.1) | 2246 (44.5) | 405 (31.3) | 4213 (38.8) | 838 (50.2) | 4834 (44.6) |
| Vocational school or short college | 182 (34.6) | 2173 (39.4) | 108 (16.6) | 884 (17.5) | 446 (34.5) | 4245 (39.1) | 280 (16.8) | 1907 (17.6) |
| Undergraduate studies | 157 (29.9) | 1066 (19.3) | 168 (25.8) | 1599 (31.7) | 386 (29.8) | 2139 (19.7) | 427 (25.6) | 3403 (31.4) |
| Graduate studies | 17 (3.2) | 87 (1.6) | 38 (5.8) | 275 (5.5) | 41 (3.2) | 174 (1.6) | 95 (5.7) | 596 (5.5) |
| Missing | 6 (1.1) | 44 (0.8) | 11 (1.7) | 43 (0.9) | 14 (1.1) | 87 (0.8) | 28 (1.7) | 87 (0.8) |
| **Occupational Status** |  |  |  |  |  |  |  |  |
| Regular employee | 231 (43.9) | 1546 (28) | 276 (42.4) | 3680 (72.9) | 568 (43.9) | 3279 (30.2) | 706 (42.3) | 7760 (71.6) |
| Part-time or temporary worker | 167 (31.8) | 1662 (30.1) | 152 (23.4) | 448 (8.9) | 411 (31.8) | 3257 (30.0) | 392 (23.5) | 1051 (9.7) |
| Business owner or family business | 16 (3.0) | 242 (4.4) | 33 (5.1) | 480 (9.5) | 39 (3.0) | 467 (4.3) | 83 (5.0) | 1019 (9.4) |
| Unemployed | 75 (14.3) | 1852 (33.6) | 127 (19.5) | 184 (3.7) | 184 (14.2) | 3431 (31.6) | 324 (19.4) | 444 (4.1) |
| Student | 4 (0.8) | 23 (0.4) | 15 (2.3) | 31 (0.6) | 10 (0.8) | 43 (0.4) | 38 (2.3) | 76 (0.7) |
| Missing | 33 (6.3) | 190 (3.5) | 48 (7.4) | 224 (4.4) | 82 (6.3) | 380 (3.5) | 125 (7.5) | 477 (4.4) |
| **Annual Income (in JPY 10,000s)**^†^ |  |  |  |  |  |  |  |  |
| 0-99 | 166 (31.6) | 3046 (55.2) | 236 (36.3) | 603 (12.0) | 409 (31.6) | 5668 (52.2) | 606 (36.3) | 1376 (12.7) |
| 100-299 | 220 (41.8) | 1414 (25.6) | 212 (32.6) | 1084 (21.5) | 540 (41.7) | 2986 (27.5) | 541 (32.4) | 2439 (22.5) |
| 300-499 | 99 (18.8) | 748 (13.6) | 149 (22.9) | 2106 (41.7) | 245 (18.9) | 1596 (14.7) | 384 (23.0) | 4509 (41.6) |
| 500-799^†^ | 13 (2.5) | 155 (2.8) | 24 (3.7) | 921 (18.3) | 34 (2.6) | 304 (2.8) | 62 (3.7) | 1842 (17.0) |
| ≥800 | - | - | 2 (0.3) | 149 (3) | - | - | 5 (0.3) | 282 (2.6) |
| Missing | 28 (5.3) | 152 (2.8) | 28 (4.3) | 184 (3.7) | 67 (5.2) | 304 (2.8) | 72 (4.3) | 379 (3.5) |
| **Region of Residence** |  |  |  |  |  |  |  |  |
| Kanto | 184 (35.0) | 1851 (33.6) | 240 (36.9) | 1688 (33.5) | 449 (34.7) | 3659 (33.7) | 621 (37.2) | 3631 (33.5) |
| Chubu | 103 (19.6) | 1093 (19.8) | 121 (18.6) | 1010 (20.0) | 254 (19.6) | 2128 (19.6) | 310 (18.6) | 2157 (19.9) |
| Kinki | 102 (19.4) | 819 (14.9) | 115 (17.7) | 708 (14.0) | 251 (19.4) | 1596 (14.7) | 292 (17.5) | 1506 (13.9) |
| Kyushu/Okinawa | 46 (8.8) | 642 (11.6) | 57 (8.8) | 606 (12.0) | 113 (8.7) | 1292 (11.9) | 145 (8.7) | 1322 (12.2) |
| Chugoku/Shikoku | 43 (8.2) | 481 (8.7) | 52 (8.0) | 458 (9.1) | 105 (8.1) | 945 (8.7) | 130 (7.8) | 986 (9.1) |
| Tohoku | 35 (6.7) | 431 (7.8) | 56 (8.6) | 395 (7.8) | 89 (6.9) | 858 (7.9) | 145 (8.7) | 845 (7.8) |
| Hokkaido | 13 (2.5) | 198 (3.6) | 10 (1.5) | 182 (3.6) | 32 (2.5) | 380 (3.5) | 25 (1.5) | 390 (3.6) |
| **Population Density and Size of Residence (in 10,000s)** |  |  |  |  |  |  |  |  |
| Non-densely inhabited area | 189 (35.9) | 1845 (33.5) | 237 (36.4) | 1705 (33.8) | 462 (35.7) | 3616 (33.3) | 608 (36.4) | 3663 (33.8) |
| < 20 | 113 (21.5) | 1186 (21.5) | 141 (21.7) | 1126 (22.3) | 281 (21.7) | 2334 (21.5) | 355 (21.3) | 2428 (22.4) |
| 20-100 | 135 (25.7) | 1379 (25.0) | 172 (26.4) | 1213 (24.0) | 329 (25.4) | 2736 (25.2) | 446 (26.7) | 2623 (24.2) |
| ≥100 | 89 (16.9) | 1105 (20.0) | 101 (15.5) | 1003 (19.9) | 223 (17.2) | 2172 (20.0) | 260 (15.6) | 2124 (19.6) |
| **Wish to get married in lifetime** |  |  |  |  |  |  |  |  |
| No | 94 (17.9) | - | 132 (20.3) | - | 232 (17.9) | - | 340 (20.4) | - |
| Yes | 402 (76.4) | - | 476 (73.1) | - | 989 (76.4) | - | 1218 (73.0) | - |
| Missing | 30 (5.7) | - | 43 (6.6) | - | 74 (5.7) | - | 110 (6.6) | - |

Extrapolated numbers are shown in 1000s.

^*^ Currently enrolled students were categorized according to their ongoing education.

^†^ ≥ JPY 500 for women; JPY= Japanese Yen.

**References**

1. National Institute of Population and Social Security Research. *The Fifteenth Japanese National Fertility Survey in 2015. Marriage Process and Fertility of Married Couples Attitudes toward Marriage and Family among Japanese Singles. Summary of the Survey Results on Married Couples/Singles.*; 2017.

2. National Institute of Population and Social Security Research. The Fourteenth Japanese National Fertility Survey in 2010 Attitudes toward Marriage and Family among Japanese Singles Highlights of the Survey Results on Singles. 2011.

3. National Institute of Population and Social Security Research. The Fourteenth Japanese National Fertility Survey in 2010 Attitudes toward Marriage and Family among Japanese Singles Highlights of the Survey Results on Married Couples. 2011.

4. Kaneko R, Sasai T, Kamano S, Iwasawa M, Mita F, Moriizumi R. Marriage Process and Fertility of Japanese Married Couples. Overview of the Results of the Thirteenth Japanese National Fertility Survey, Married Couples. *Japanese J Popul*. 2008;6(1).

5. Kaneko R, Sasai T, Kamano S, Iwasawa M, Mita F, Moriizumi R. Attitudes toward Marriage and the Family among Japanese Singles. Overview of the Results of the Thirteenth Japanese National Fertility Survey, Singles. *Japanese J Popul*. 2008;6(1).

6. National Institute of Population and Social Security Research. [The Twelfth Japanese Fertility Survey in 2002. Summary of Results]. http://www.ipss.go.jp/ps-doukou/j/doukou12/doukou12.asp. Accessed June 28, 2018.

7. Takahashi S, Kaneko R, Sato R, et al. The Eleventh Japanese National Fertility Survey in 1997. Attitudes toward Marriage and the Family among the Unmarried Japanese Youth. *J Popul Soc Secur*. 2003;1(1).

8. Takahashi S, Kaneko R, Sato R, et al. The Eleventh Japanese National Fertility Survey in 1997. Marriage and Fertility in Present-Day Japan. *J Popul Soc Secur*. 2003;1(1).

9. Atoh M, Takahashi S, Nakano E, Watanabe Y, Kojima H, Kaneko R. [Trends in marriage and fertility in Japan: major findings from the Tenth Japanese National Fertility Survey]. *Jinko Mondai Kenkyu*. 1993;49(3):1-28.

10. Atoh M, Nakano E, Otani K, Kaneko R. [Marriage and fertility in present-day Japan: major findings of the Ninth Japanese National Fertility Survey]. *Jinko Mondai Kenkyu*. 1988;(187):1-28.

11. StataCorp LP. *Stata Survey Data Reference Manual. Release 13*. College Station, TX; 2013.

12. Bell BA, Onwuegbuzie AJ, Ferron JM, Jiao QG, Hibbard ST, Kromrey JD. Use of Design Effects and Sample Weights in Complex Health Survey Data: A Review of Published Articles Using Data From 3 Commonly Used Adolescent Health Surveys. *Am J Public Health*. 2012;102(7):1399-1405. doi:10.2105/AJPH.2011.300398.

13. Ministry of Internal Affairs and Communications. Statistics Japan. Statistics Bureau. Population Census. http://www.stat.go.jp/english/data/kokusei/index.html. Published 2015. Accessed June 25, 2018.

14. Statistics Japan. Statistics Bureau, Ministry of Internal Affairs and Communications. What is a Densely Inhabited District? http://www.stat.go.jp/english/data/chiri/did/1-1.html. Accessed June 25, 2018.
